# Application of Diffusion Weighted Imaging Techniques for Differentiating Benign and Malignant Breast Lesions

**DOI:** 10.3389/fonc.2021.694634

**Published:** 2021-06-21

**Authors:** Muzhen He, Huiping Ruan, Mingping Ma, Zhongshuai Zhang

**Affiliations:** ^1^ Shengli Clinical Medical College of Fujian Medical University, Fuzhou, China; ^2^ Department of Radiology, Fujian Provincial Hospital, Fuzhou, China; ^3^ Siemens Healthcare Ltd., Shanghai, China

**Keywords:** breast lesion, magnetic resonance imaging, apparent diffusion coefficient, intra-voxel incoherent motion, diffusion kurtosis imaging

## Abstract

To explore the value of apparent diffusion coefficient (ADC), intravoxel incoherent motion (IVIM), and diffusional kurtosis imaging (DKI) based on diffusion weighted magnetic resonance imaging (DW-MRI) in differentiating benign and malignant breast lesions. A total of 215 patients with breast lesions were prospectively collected for breast MR examination. Single exponential, IVIM, and DKI models were calculated using a series of b values. Parameters including ADC, perfusion fraction (*f*), tissue diffusion coefficient (*D*), perfusion-related incoherent microcirculation (D*), average kurtosis (MK), and average diffusivity (MD) were compared between benign and malignant lesions. ROC curves were used to analyze the optimal diagnostic threshold of each parameter, and to evaluate the diagnostic efficacy of single and combined parameters. ADC, *D*, MK, and MD values were significantly different between benign and malignant breast lesions (P<0.001). Among the single parameters, ADC had the highest diagnostic efficiency (sensitivity 91.45%, specificity 82.54%, accuracy 88.84%, AUC 0.915) and the best diagnostic threshold (0.983 μm^2^/ms). The combination of ADC and MK offered high diagnostic performance (sensitivity 90.79%, specificity 85.71%, accuracy 89.30%, AUC 0.923), but no statistically significant difference in diagnostic performance as compared with single-parameter ADC (P=0.268). The ADC, *D*, MK, and MD parameters have high diagnostic value in differentiating benign and malignant breast lesions, and of these individual parameters the ADC has the best diagnostic performance. Therefore, our study revealed that the use of ADC alone should be useful for differentiating between benign and malignant breast lesions, whereas the combination of MK and ADC might improve the diagnostic performance to some extent.

## Introduction

Breast cancer is the most common cancer in women. In 2018 there were approximately 2.1 million newly diagnosed female breast cancer cases worldwide, accounting for a quarter of female cancer cases (1). In China the incidence of breast cancer is relatively low, but since 1990 the incidence of breast cancer has increased rapidly, especially in urban areas (2, 3). Survival rates for breast cancer are greatly improved by early diagnosis. The main techniques used for identification of breast lesions are ultrasound, mammography, and magnetic resonance imaging (MRI). MRI offers better sensitivity and specificity than mammography and ultrasonography, especially for lesions in dense breasts (4, 5).

MRI can not only analyze the nature of the lesion through morphological features, but also obtain a variety of quantitative parameters using functional imaging sequences for more objective evaluation and diagnosis (6). Diffusion weighted MRI (DW-MRI), which indirectly reflects the degree of tissue differentiation and the integrity of cell membranes, is routinely used to improve the accuracy of differential breast lesion diagnoses (7–9).

The single exponential model is useful to distinguish benign from malignant breast lesions, and has been most widely used in clinical practice because of its short scanning time and simple post-processing (8, 10, 11). The ADC model requires two b-values to fit the curve. Many studies have shown that ADC has a certain significance in the identify of benign and malignant breast lesions (8, 9). The intravoxel incoherent motion (IVIM) model is first proposed by Bihan et al. (12) and has been reported to have good diagnostic performance for the diagnosis of pancreatic ductal adenocarcinoma (13). When b-value is low (≤200 s/mm^2^), tissue diffusion is affected by microcirculation perfusion. As the b-value increases, the proportion of microcirculation perfusion is gradually reduced and it probably reflects the diffusion of water molecules in the tissue (14). However, the disadvantage of a high b-value is that it can reduce the signal-to-noise ratio. Therefore, in IVIM studies the b-value usually ranges from 0 to 1000 s/mm^2^, and four to more than 10 different b-values are required to obtain perfusion fraction (*f*), tissue diffusion coefficient (*D*) and perfusion-related incoherent microcirculation (D*) values (14, 15). Liu et al. (16) have shown that when b<200 s/mm^2^, the attenuation speed of malignant lesions is significantly faster than that of normal breast tissue and benign lesions.

In the traditional DWI model, the diffusion of water molecules follows a Gaussian distribution, so the b-value affects the ADC value. In the diffusional kurtosis imaging (DKI) model first proposed by Jensen et al. (17), when the b-value is high (>1000 s/mm^2^) the diffusion of water molecules follows a non-Gaussian distribution, the DKI model is more accurate at assessing the diffusion of water molecules in a lesion (17–19). In recent years, extended DWI models based on different b-values, including IVIM and DKI, have been used for the identification of tumors in liver (20), prostate (21, 22), thyroid (23) and brain (24). However, there have been few studies combining ADC, IVIM, and DKI values for use in the differentiation of benign and malignant breast lesions.

## Materials and Methods

### Patients

The institutional ethics committee of our hospital approved this prospective study, and informed consent was provided by each patient. Patients with suspicious breast lesions from June 2019 to October 2020 were prospectively collected. Inclusion criteria: (1) No puncture, biopsy, radiotherapy, or chemotherapy were performed before MRI examination. (2) No contraindications to MRI examination. (3) All patients underwent plain MRI and multi-b-value DWI. (4) There was complete biopsy or surgical pathology. Exclusion criteria: (1) The solid part of the lesion was too small to delineate the ROI. (2) Image quality was poor and did not meet the post-processing requirements.

### Scanning Method

All MR examinations were performed in a 3.0T MR (MAGNETOM Prisma, Siemens Healthcare, Erlangen, Germany) with 18-channel dual breast-dedicated phase-controlled surface coil. All patients were scanned in the prone position, with breasts naturally suspended in the coil. The sequences included T1WI (TR/TE=6.03/2.82 ms, thickness = 0.9 mm, number of slices = 160, bandwidth = 300 Hz/Px, FOV read = 340 mm, FOV phase = 100%, matrix size = 403×448), Fat saturation T2WI (TR/TE = 3730/69 ms, thickness = 4 mm, number of layers = 35, bandwidth = 246 Hz/Px, FOV read = 340 mm, FOV phase = 100%, matrix size = 384×384, averages = 2, concatenations = 2) and dynamic contrast enhanced MRI (DCE-MRI) (TR/TE = 4.03/1.33ms, thickness = 1.5 mm, number of slices = 112, bandwidth = 1120 Hz/Px, FOV read = 350 mm, FOV phase = 100%, matrix size = 259×320, Measurements 36, scan time = 343 s). The parameters of multiple b-value DWI sequences were TR/TE = 5700/62 ms, layer thickness = 4 mm, number of layers = 35, bandwidth = 2024 Hz/Px, FOV read = 340 mm, FOV phase = 60%, matrix size = 114×190; b-values = 0, 30, 50, 80, 120, 160, 200, 500, 1000, 1500, 2000 s/mm^2^, averages = 1, 1, 1, 1, 1, 1, 1, 1, 2, 2, 3; scan time = 308 s.

### Data Analysis

For each model, all DWI data were fitted pixel by pixel using a prototype software (Body Diffusion Toolbox, Siemens Healthcare, Erlangen, Germany), and the relevant parameter maps of ADC, IVIM, and DKI were obtained. Parameters including perfusion fraction (*f*), tissue diffusion coefficient (*D*), perfusion-related incoherent microcirculation (D*), mean kurtosis (MK), and mean diffusivity (MD) were calculated. For the single exponential model, two b-values (0 and 1000 s/mm^2^) were chosen with the equation. S(b)=S(0)×exp(-b× ADC) (1, 25). For the IVIM model, a total of nine b-values (0, 30, 50, 80, 120, 160, 200, 500, and 1000 s/mm^2^) were used for data calculation using the classic two-step calculation method (26). The applied equation was: S (b)/S (0) = (1-*f*) × exp(-b·*D*) + *f* × exp[-b·(D* + *D*)] (2, 13). The parameter *D* was obtained using the data of b > 400 s/mm^2^. D* and *f* over all b values was calculated by a nonlinear regression algorithm, while keeping *D* constant (27). Five high b-values (0, 500, 1000, 1500, and 2000 s/mm^2^) were selected for the DKI model, using the equation (17) S(b)=S (0) ×exp (-b× MD) + 1/6× b^2^× MD^2^× MK) (3). For all the formulas above, b is the diffusion-sensitive gradient factor, S(0) is the tissue signal intensity in the voxel when b=0 s/mm^2^, and S(b) is the signal strength of the tissue within the element when b>0 s/mm^2^. The mean value of signal intensity distribution within the region of interest (ROI) was calculated for each b value. Then, the mean signal intensities of b values in Eqs. (2) and (3) were fitted with the least square method using the Levenberg-Marquardt algorithm. The upper and lower limits of f and D* were 0%-40% and 0-50×10^−3^ mm^2^/s respectively by referring to the range of each parameter in an earlier report (28). The goodness of fit in both the IVIM and DKI fittings was assessed by the coefficient of determination R2(R2 = 1−ESS/TSS), where ESS and TSS is the sum of the squared errors between the data points and IVIM/DKI fitting curve, and the sum of the squared differences between the data points and the mean value of all data points, respectively. The pixel was excluded if its R2 value was < 0.8 (29).

### ROI Delineation and Parameter Calculation

**Figure 1:**
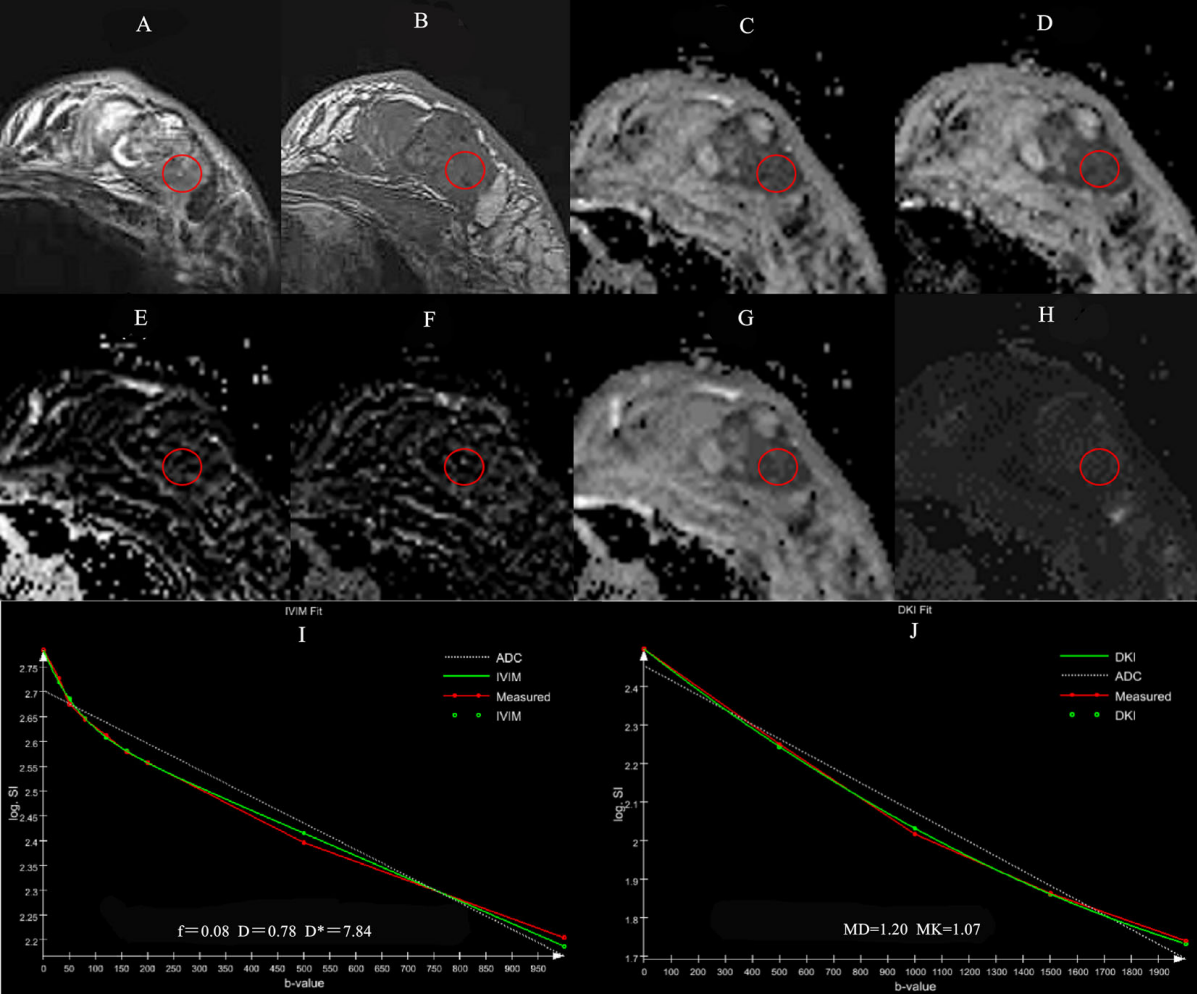
A 56-year-old female patient with the left breast mass. The mass is located in outer quadrant of left breast (red round ROI), showing heterogeneous hyperintensity on TIRM **(A)**, hypointensity on T1WI **(B)**, ADC **(C)**, *D* **(D)** maps, isointensity on *f* **(E)**, D* **(F)** maps, hypointensity on MD **(G)** and hyperintensity on MK **(H)** maps. Graphs show signal intensity *vs.* b value fits in single pixels of invasive ductal carcinoma of the breast with the IVIM **(I)** and DKI **(J)** models.

The ROI was measured by two radiologists with 10 years and 2 years of experience in breast imaging diagnosis. They read the images independently without knowing the pathological results and measured twice on the ADC image (b=1000 s/mm^2^) at the largest level of the solid component of the lesion, avoiding obvious necrosis, cystic and liquefaction areas by referring to fat saturation T2WI and DCE-MRI imaging. The averaged ROI was then overlaid on the other parameter maps to obtain their corresponding parameters (**Figure 1**).

### Statistical Methods

The Shapiro-Wilk normality test and the Levene variance homogeneity test were performed for all continuous variables. Values are described as mean ± standard deviation, and either a t-test or Mann-Whitney U test was used for comparisons between groups. The receiver operating characteristic (ROC) was used to evaluate the diagnostic efficacy of each parameter. The stepwise backward logistic regression method was used to fit multiple parameters (P<0.1), and the parameters that were retained in the equation were combined to generate predicted probabilities for ROC curve evaluation. GraphPad Prism software (version 7.0) was used to draw the box plots, and SPSS (version 22.0) and R (version 3.6.0) software were used for statistical analysis. Significance was defined as p<0.05. The DeLong test was used to compare diagnostic efficiency across different parameters.

Consistency of the parameters was evaluated by comparing correlation factors within and between groups. Consistency within a group was evaluated by comparing two measurements by the same radiologist, and consistency between groups was evaluated by comparing the first measurement of each radiologist. When the interclass correlation coefficient (ICC) was greater than 0.75, consistency was considered good; between 0.50 and 0.75, fair; less than 0.50, very poor.

## Results

### Clinical Data

A total of 202 female patients were enrolled. The average age of 54 patients in the benign group was 43.8 ± 9.2 years (range 28–62), and the average age of 148 patients in the malignant group was 52.1 ± 11.0 years (range 27–80). The age difference between the two groups was statistically significant (P<0.001).

### Pathological Results

Among the 202 patients there were 215 lesions, 63 of which were benign (including 15 adenopathy, 45 fibroadenoma, and 3 abscesses), and 152 of which were malignant (including 16 carcinomas in situ, 135 invasive carcinomas, and 1 adenoid cystadenocarcinoma).

### Quantitative Parameters

**Figure 2:**
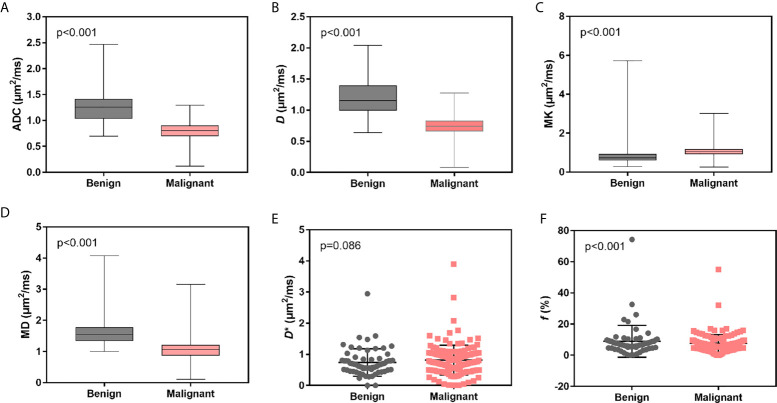
Box and scatter plots for the average distribution of ADC, *D*, MK, MD, D*, and *f* of benign *vs.* malignant breast lesions. **(A–D)** Average value of ADC, *D*, MK and MD. **(E, F)** Average values of D* and *f*.

ADC, *D*, MK, and MD values were statistically significant in the identification of benign and malignant breast lesions (P<0.001), while *f* and D* were not (P>0.05) (**Table 1**). The average values of ADC, *D*, and MD were greater in the benign lesion group than in the malignant lesion group, while the average MK value was smaller (**Table 1** and **Figure 2**).

**Table 1:** Statistical result of various quantitative parameters in distinguishing benign and malignant breast lesions.

	Benign Lesions (n = 63)	Malignant Lesions (n = 152)	Z value	P value
ADC (μm^2^/ms)	1.26 ± 0.21 (1.03-1.41)	0.80 ± 0.09 (0.70-0.90)	-9.570	<0.001
*f* (%)	6.85 ± 0.54 (5.03-9.45)	6.93 ± 0.71 (4.85-8.81)	-0.322	0.748
*D* (μm^2^/ms)	1.15 ± 0.22 (0.99-1.39)	0.74 ± 0.17 (0.66-0.83)	-9.432	<0.001
D* (μm^2^/ms)	6.71 ± 0.41 (5.21-8.40)	7.73 ± 0.73 (5.25-10.31)	1.717	0.086
MK (μm^2^/ms)	0.74 ± 0.19 (0.6-0.93)	1.05 ± 0.29 (0.92-1.17)	6.200	<0.001
MD (μm^2^/ms)	1.55 ± 0.56 (1.34-1.78)	1.07 ± 0.23 (0.87-1.21)	-9.100	<0.001

### Diagnostic Efficiency

**Figure 3:**
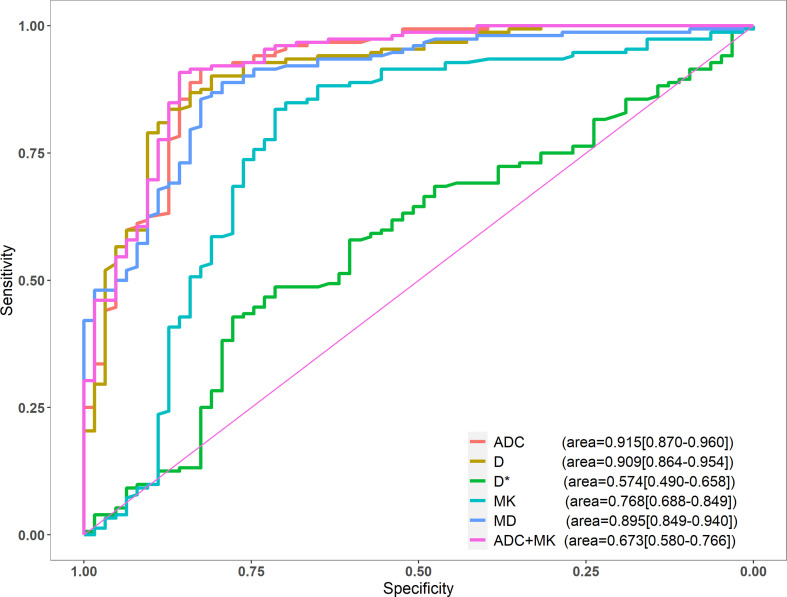
ROC curves of ADC, *D*, D*, MK, and MD.

Among the single-parameter indicators, ADC achieved the highest sensitivity (91.45%), specificity (82.54%), and accuracy (88.84%). The area under the ROC curve (AUC) was 0.915, and the critical value for diagnosis was 0.983 μm^2^/ms. After logistic regression analysis, the combined application of ADC and MK outperformed ADC on most measures, demonstrating higher specificity (85.71%), accuracy (89.30%), and AUC (0.923) (**Table 2**). AUCs for all single parameters, as well as for ADC + MK, are shown in **Figure 3**.

**Table 2:** The diagnostic performance of single and combined parameters.

	Threshold	AUC (95%CI)	Sensitivity	Specificity	Accuracy
ADC (μm^2^/ms)	<0.983	0.915 (0.870-0.960)	91.45% (139/152)	82.54% (52/63)	88.84% (191/215)
*D *(μm^2^/ms)	<0.952	0.909 (0.864-0.954)	90.13% (137/152)	80.95% (51/63)	87.44% (188/215)
D* (μm^2^/ms)	>0.873	0.574 (0.490-0.658)	42.76% (65/152)	77.78% (49/63)	53.02% (114/215)
MK (μm^2^/ms)	>0.864	0.768 (0.688-0.849)	83.55% (127/152)	71.43% (45/63)	80.00% (172/215)
MD (μm^2^/ms)	<1.297	0.895 (0.849-0.940)	88.82% (135/152)	79.37% (50/63)	86.05% (185/215)
ADC+MK	/	0.923 (0.881-0.964)	90.79% (138/152)	85.71% (54/63)	89.30% (192/215)

The DeLong test showed that the diagnostic efficacy of ADC was better than that of MK and D* (P<0.001), and there was no statistical difference between *D* and MD (P=0.524, 0.180). There was no significant difference in diagnostic efficacy between ADC alone as compared to ADC + MK (P=0.268).

### Consistency Testing

ICC values for ADC, *D*, MK and MD measurements were all greater than 0.75, with good consistency of the inter- and intra-reader reproducibility. The ICC values for *f* measurement (inter-reader) was 0.675, and *f* and D* measurements (intra-reader) was 0.724.

## Discussion

In this study, we find that the ADC, *D*, MK and MD values of different DWI techniques have high diagnostic value in differentiating benign and malignant breast lesions. Of these parameters, ADC had the best diagnostic performance, and the combined application of ADC and MK values achieved even higher diagnostic accuracy. A meta-analysis based on 13,847 lesions showed that ADC was meaningful in the differentiation of benign and malignant lesions, and recommended using an ADC value of 1.0 μm^2^/ms as the threshold (9). In the present study, we found that if ADC <0.983 μm^2^/ms was used as the threshold, the sensitivity was 91.45%, specificity was 82.54%, accuracy was 88.84%, and AUC was 0.915. Two b-values (0 and 1000 s/mm^2^) were selected and the results showed that the average ADC of benign lesions of this group was significantly lower than that benign ones of meta-analysis, which is likely due to the mainly inclusion of fibroadenoma in the benign group of this study; fibrosis is known to reduce ADC values (25).

According to previous studies, the IVIM model should include b-values greater than 200 s/mm^2^ (14). In the present study a total of nine b-values were selected, seven of which were less than 200 s/mm^2^ to ensure accurate reflection of the diffusion of water molecules and blood microcirculation perfusion. In this analysis only *D* was able to reliably differentiate between benign and malignant breast lesions. *D* reflects the true diffusion of water molecules after removing the effects of microcirculation perfusion, and the *D* value of malignant lesions is significantly lower than that of benign ones, as we and others have shown (16, 26, 30). *f* represents the ratio between microcirculation perfusion and overall diffusion. In a study by Liu et al. (16), the *f* value of malignant lesions was found to be significantly higher than that of benign lesions, which was thought to be related to the higher microcirculation blood volume of malignant tumors. In the present study, the average value of *f* was slightly higher in the malignant group, but the difference was not statistically significant. This may be due to the poor repeatability of *f* values between different observers and different machines (31). D* represents the perfusion-related diffusion of microcirculation within the voxel, which is easily affected by neighboring structures and motion artifacts. As such, it is not known to be a good indicator of benign *vs.* malignant lesions (16, 30). Of the three IVIM parameters, we would recommend only *D* for use in differentiating benign and malignant breast lesions.

According to a preliminary study by Nogueira et al. (19), the DKI model needs to contain high (>200 s/mm^2^) b-values. This study used five b-values (0, 500, 1000, 1500 and 2000 s/mm^2^), and showed that MD of the malignant group was significantly lower than that of the benign group. This level of diagnostic efficiency is consistent with the results of other studies (18, 32). MK takes into account the heterogeneity and restriction of diffusion, and therefore reflects the complexity of biological tissues (24). Malignant lesions tend to have higher MK values than benign lesions due to structural heterogeneity, high cell density, interstitial vascular proliferation, and complex tissue structure, which was demonstrated in this and other studies (18, 32).

In this study, the diagnostic power of the ADC value was slightly higher than that of the DKI model. However, there was no significant difference between the two groups, which is consistent with the meta-analysis of Li et al. (32). The combined parameters of ADC and MK had the highest diagnostic efficiency, but there was no statistically significant difference between the combined parameters and single-parameter ADC. Taking into account the increased cost of combined parameter scanning and processing times, the single-parameter ADC value is more suitable for routine clinical applications.

There are limitations to this study. First, to ensure objectivity of data selection, ROIs were selected on an ADC map with b=1000 s/mm^2^ and then copied to other parameter maps. However, this method may have allowed for the inclusion of images with poor signal-to-noise ratio, so the repeatability of IVIM and DKI parameters in this group is likely to be poor. Second, ROI measurements on one or even several selected sections of the tumor cannot reflect the tumor heterogeneity comprehensively. Therefore, the whole-tumor histogram analysis may be a more integrated method to investigate the histopathologic basis. Finally, some scholars believe that there is a correlation between IVIM or DKI parameters and prognostic factors of breast cancer, such as tumor size, nuclear grade, biological markers, and metastatic lymph nodes (26, 32, 33), but we did not investigate the correlation between them and need to be further improved in future research.

In conclusion, in the single parameter index of each DW-MRI model in this study, ADC was most valuable in the differential diagnosis of benign and malignant breast lesions. Although the combined application of ADC and MK values can achieve higher diagnostic efficacy than ADC alone, the difference is not statistically significant. Since the ADC image offers high signal-to-noise ratio, good data repeatability, and has the advantages of simple and quick detection, the single index model is worthy of further promotion in clinical applications.

## Data Availability Statement

The raw data supporting the conclusions of this article will be made available by the authors, without undue reservation.

## Ethics Statement

The studies involving human participants were reviewed and approved by the institutional ethics committee of Fujian Provincial Hospital. The patients/participants provided their written informed consent to participate in this study.

## Author Contributions

MM and ZZ contributed to conception and design of the study. HR organized the database and wrote the first draft of the manuscript. MH performed the statistical analysis and wrote sections of the manuscript. All authors contributed to the article and approved the submitted version.

## Funding

This work was supported by China International Medical Foundation (Grant number:Z-2014-07-1912-23), and the Natural Science Foundation of Fujian Province, China (Grant number:2020J011057).

## Conflict of Interest

Author ZZ was employed by the company Siemens Healthcare Ltd.

The remaining authors declare that the research was conducted in the absence of any commercial or financial relationships that could be construed as a potential conflict of interest.
